# Telestroke Centers as an Option for Addressing Geographical Disparities in Access to Stroke Care in South Carolina, 2013

**DOI:** 10.5888/pcd12.150418

**Published:** 2015-12-24

**Authors:** Marsha Samson, Tushar Trivedi, Khosrow Heidari

**Affiliations:** Author Affiliation: Tushar Trivedi, Cancer Prevention and Control Program, Arnold School of Public Health, University of South Carolina, Columbia, South Carolina; Khosrow Heidari, South Carolina Department of Health and Environmental Control, Columbia, South Carolina.

## Abstract

Telestroke centers can increase access to proper and timely diagnosis and treatment of stroke, especially for rural populations, thereby reducing disability and death. Census tract information was used to map primary stroke centers geographically and to identify areas that would benefit from additional access to medical care via telestroke centers (health care facilities that provide information on stroke care from a distance). Results indicate that in 2013, approximately half of the South Carolina population did not have access to a primary stroke center within a 30-minute drive of their home, and 30% did not have access within 60 minutes. Increasing access to prompt evaluation, diagnosis, and treatment of stroke and improving long-term quality of life requires the addition of telestroke centers in areas without primary stroke centers and examination of the effects of these centers on stroke incidence and mortality in South Carolina.

## Objective

Stroke is a major public health issue and the leading cause of long-term disability (1,2) in the United States. Timely evaluation, diagnosis, and treatment are critical to reducing incidence and death related to acute ischemic stroke (3). We used geographic information system (GIS) analysis to identify regions of South Carolina with access to stroke centers and regions without such access. Negative stroke outcomes in the southeastern part of the United States may be directly related to the lack of easily accessible primary stroke treatment facilities. Primary stroke centers (PSCs) are hospital centers equipped with resources and processes to treat people with acute stroke (4). In underserved areas, telestroke centers (health care facilities that provide information on stroke care from a distance through electronic communication) reduce health inequities and complement PSCs by ensuring early care for stroke victims who live more than 30 to 60 minutes from a PSC (5). Age-adjusted death rates from stroke are higher in South Carolina than in the nation overall (6). Our objective was to examine how telestroke centers can address geographic disparities in access to stroke care in South Carolina.

## Methods

We used census tract information and GIS mapping technology to calculate areas within 30-minute and or 60-minute drive times to PSCs or telestroke centers and the proportion of the state population living within those drive times. In addition to PSCs in South Carolina, we also considered PSCs in nearby states (North Carolina and Georgia) that were within 30- or 60-minute drive times for South Carolina residents. Using data from vital statistics, we also identified the 24 counties in South Carolina with the highest age-adjusted death rate for stroke in the state; we overlaid drive time areas on these counties. We used GIS maps to illustrate geographic disparities in access to stroke care offered at certified PSCs and the contribution of telestroke centers to addressing these geographic disparities.

## Results

**Figure 1:**
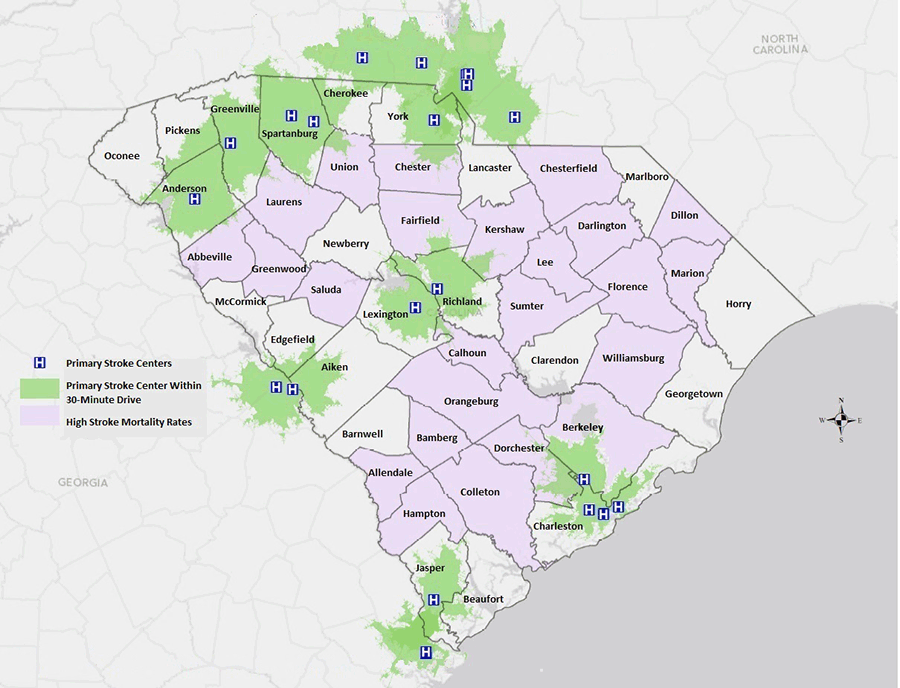
South Carolina primary stroke centers (PSCs) and the population within a 30-minute drive time (54% of the South Carolina population lives within a 30-minute drive of a PSC).

**Figure 2:**
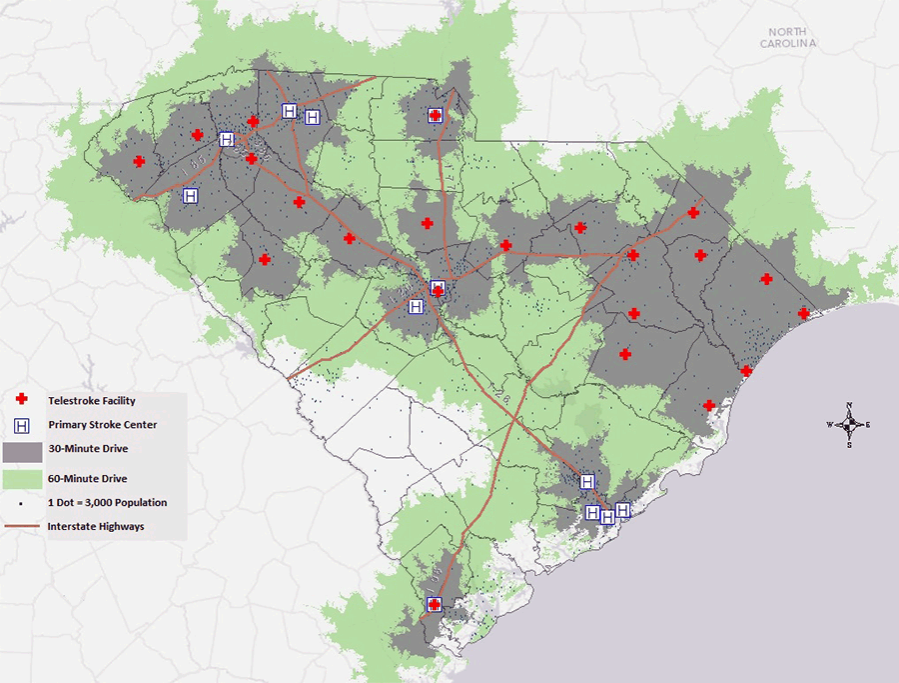
South Carolina primary stroke centers and telestroke centers and the population within a 30-minute or 60-minute drive time (95% of the South Carolina population live within a 60-minute drive of a primary stroke center or a telestroke center, and 76% live within a 30-minute drive).

Most South Carolina counties had high death rates for stroke in 2013 (Figure 1). Those counties had limited or no access to a PSC within a 30-minute or 60-minute drive time. However, telemedicine facilities such as telestroke centers could increase access to stroke care in these areas. Most primary stroke centers were located in the northern half of the state (in Anderson, Greenville, and Spartanburg) or the eastern part of the Low Country (coastal South Carolina) (in Charleston). Approximately 54% of the South Carolina population lives within a 30-minute drive of a PSC, and 77% lives within a 60-minute drive (Figure 1). By including telestroke centers within 30-minute or 60- minute drive times, residents of South Carolina had access to significantly more stroke care centers (76% within 30 minutes and 95% within 60 minutes) (Figure 2). Since 2008, despite a significant increase in the distribution of stroke resources within the state from the addition of telestroke centers, central South Carolina and the western part of the Low Country still did not have access to quality stroke care.

## Discussion

The shortage of specialized stroke treatment facilities in South Carolina results in huge geographical disparities in access to stroke care and in the quality of stroke care. The need for neurologists in South Carolina is projected to increase by 2025, and rural populations will face greater disparities in care (7). Observational studies showed that telemedicine continues to have a positive impact on health when compared with in-person visits (8). Local and state health partners could provide telestroke centers through community partnerships, program planning, and appropriate resource allocation. This study shows that many areas in South Carolina lack timely access to PSCs. This information can serve as a catalyst for initiating new discussions and informing the policy decision-making process. Expansion of stroke services in regions that lack such services requires informing health care providers and administrative personnel in rural hospitals about telestroke centers and showing that they are a viable and low-cost resource for providing quality stroke care. Having under-resourced rural hospitals without stroke care facilities join with stroke care centers of excellence would make specialized, streamlined stroke services available in disparate geographical areas. 
